# Factors related to medical students’ and doctors’ attitudes towards older patients: a systematic review

**DOI:** 10.1093/ageing/afx058

**Published:** 2017-05-02

**Authors:** Rajvinder Samra, Tom Cox, Adam Lee Gordon, Simon Paul Conroy, Mathijs F G Lucassen, Amanda Griffiths

**Affiliations:** 1 School of Health, Wellbeing and Social Care, The Open University, Milton Keynes, United Kingdom of Great Britain and Northern Ireland; 2 Centre for Sustainable Working Life, School of Business, Economics and Informatics, Birkbeck University of London , London, United Kingdom of Great Britain and Northern Ireland; 3 Division of Medical Sciences and Graduate Entry Medicine, University of Nottingham , Nottingham, United Kingdom of Great Britain and Northern Ireland; 4 Department of Health Sciences, University of Leicester , Leicester, Leicestershire, United Kingdom of Great Britain and Northern Ireland; 5 Department of Psychological Medicine, University of Auckland , Auckland, New Zealand; 6 Division of Psychiatry and Applied Psychology, School of Medicine, University of Nottingham , Nottingham, United Kingdom of Great Britain and Northern Ireland

**Keywords:** physician, medical student, attitude, older adult, systematic review

## Abstract

**Background:**

studies have sought to identify the possible determinants of medical students’ and doctors’ attitudes towards older patients by examining relationships with a variety of factors: demographic, educational/training, exposure to older people, personality/cognitive and job/career factors. This review collates and synthesises these findings.

**Methods:**

an electronic search of 10 databases was performed (ABI/Inform, ASSIA, British Nursing Index, CINAHL, Informa Health, Medline, PsycINFO, Science Direct, Scopus, and Web of Science) through to 7 February 2017.

**Results:**

the main search identified 2,332 articles; 37 studies met the eligibility criteria set. All included studies analysed self-reported attitudes based on correlational analyses or difference testing, therefore causation could not be determined. However, self-reported positive attitudes towards older patients were related to: (i) intrinsic motivation for studying medicine, (ii) increased preference for working with older patients and (iii) good previous relationships with older people. Additionally, more positive attitudes were also reported in those with higher knowledge scores but these may relate to the use of a knowledge assessment which is an indirect measure of attitudes (i.e. Palmore's Facts on Aging Quizzes). Four out of the five high quality studies included in this review reported more positive attitudes in females compared to males.

**Conclusion:**

this article identifies factors associated with medical students’ and doctors’ positive attitudes towards older patients. Future research could bring greater clarity to the relationship between knowledge and attitudes by using a knowledge measure which is distinct from attitudes and also measures knowledge that is relevant to clinical care.

## Introduction

The rapidly ageing population has been associated with a growth in the number of older people with frailty and complex comorbidities who present to healthcare services. To meet this challenge, all doctors need to possess the necessary knowledge, skills and behaviours to care for older patients [**1**]. Recent calls for action have highlighted the need to foster positive attitudes towards older patients and caring for them [**2**]. Attempts to develop medical students’ or doctors attitudes’ towards older patients commonly involve interventions which focus on improving knowledge about ageing or older patients [**3**, **4**]. However, a recent review identified that knowledge-based interventions are unsuccessful at improving attitudes towards older patients [**4**] and, although the factors underpinning positive attitudes towards older patients have been systematically reviewed for nurses [**5**], no similar work has been done to collate the findings for medical students and doctors.

This article sets out to address this shortfall by systematically reviewing studies on medical students’ and doctors’ attitudes towards older patients, and the relationship of such attitudes to: demographic factors, education and training-related factors, exposure to older people, personality and cognitive factors and job and career factors. Identifying the factors associated with attitudes may help in the future design and delivery of interventions to foster positive attitudes, in order to ensure the medical workforce are adequately prepared to care for the growing number of older patients.

## Method

### Eligibility criteria and search strategy

Studies were included if they quantitatively measured and reported attitudes toward older patients in medical doctors or medical students. Moreover studies needed to have conducted correlational analyses or difference testing of scores on a measure of attitudes towards older patients with any other variable, and had to be published in English in a peer-reviewed journal. The review protocol, including the inclusion and exclusion criteria, is available online as Supplementary Data (Appendix A).

We combined search terms and used Boolean search in the title or abstract to search the following: [physicians, medical students plus synonyms] AND [older people or older patients, plus synonyms] AND [attitudes or beliefs, plus synonyms]. An example of our search strategy and syntax is available as Supplementary Data online (Appendix B). The following databases were searched: ABI/Inform, ASSIA, British Nursing Index, CINAHL, Informa Health, Medline, PsycInfo, Science Direct, Scopus and Web of Science. The main search was conducted in January 2016 and produced 2,332 hits once the duplicates were removed. A second search was conducted on 7 February 2017 to update the review, and this produced 90 hits after duplicates were removed.

### Study identification and selection criteria

**Figure 1.**
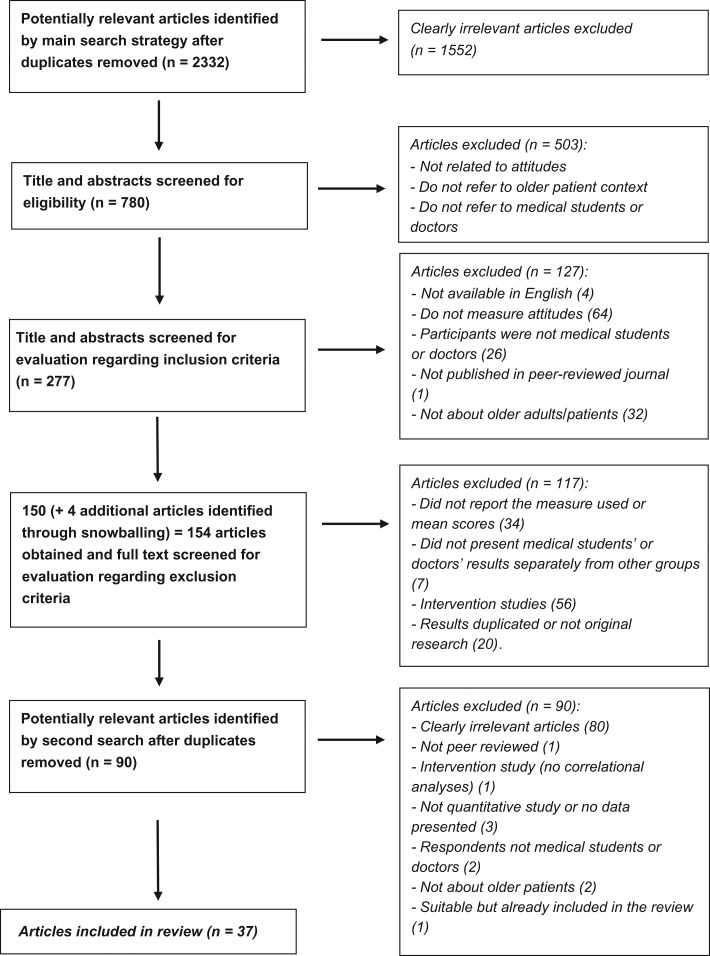
Flowchart of study inclusion and exclusion

Initial screening of the 2,332 search results from the main search removed 1,552 articles which did not address the general topic in their title or abstract and the remaining 780 articles were scanned for eligibility by R.S. Following this, 503 articles were removed because they did not measure attitudes, did not address attitudes towards older patients or include doctors or medical students as the participant group. The inclusion and exclusion criteria were applied to 277 articles. Four articles were identified by checking the reference lists at this stage as part of a snowballing exercise to identify missed articles. After application of the inclusion and exclusion criteria, 37 remained for final review. The identification of articles is illustrated in the flowchart in Figure 1. R.S. carried out the selection process and discussed uncertainties about inclusion/exclusion with an independent researcher until they reached agreement on decisions. In total, 10% of the original 2,332 articles were independently checked (by M.L.) for inclusion and exclusion. Differences and uncertainty in judgement between R.S. and M.L. took place in 1.3% of search results (*n* = 3) which were discussed until a consensus was reached. The second search (conducted on 7 February 2017) designed to update the review produced 90 hits. R.S. and M.L. both independently reviewed titles and abstracts according to the inclusion criteria and found no new articles to include.

### Quality assessment and extraction

The quality of studies were assessed using the Evaluation Tool for Quantitative Research Studies [6] and from the criteria identified in the Strengthening the Reporting of Observational Studies (STROBE) statement [**7**]. The data were not well suited for a meta-analysis due to the heterogeneity of measures employed. Details of data extraction are included in the study protocol in the Supplementary Data online (Appendix A).

## Results

Overall, 37 studies met the eligibility criteria for the present review [**8**–44]. Table 1 presents a summary of included studies.

**Table 1.** Summary of included studies

Study	Sample and setting	Attitude assessment and score range	Mean attitude score	Quality^a^
Beall *et al.* (1991) [**8**]	30 Doctors, USA	Kogan's ATOP+ [**48**]: Range 34(neg)± to 204(pos)±	167.7	Low
Belgrave *et al.* (1982) [**9**]	120 1 MS^b^, USA	Palmore's Bias Score [**50**]: Range 0(pos) to 15(neg)	5.3	Medium
Cammer-Paris *et al.* (1997) [**10**]	330 1 MS, three cohorts: 1986 (*n* = 109), 1991 (*n* = 105), 1994 (*n* = 116), USA	ASD^c^ [**47**]: Range 32(pos) to 224(neg)	1986 = 129.5; 1991 = 134.7, 1994 = 126.5	High
Cheong *et al*. (2009) [11]	342 1 MS and 3 MS, Singapore	Kogan's ATOP [**48**]: Range 34(neg) to 204(pos)	1 MS = 135.2; 3 MS = 138.2	Medium
Chua *et al.* (2008) [12]	244 1 MS, Singapore	UCLA GAS^d^ [**35**]: Range 1(neg) to 5(pos)	3.6	Medium
Chumbler* et al.* (1996) [**13**]	481 2 MS and 3 MS, USA	Locally developed scale: Satisfaction subscale range 2(neg) to 14(pos); effectiveness subscale range 4(pos) to 28(neg)	Satisfaction = 3.9; effectiveness = 7.9	High
Chumbler and Ford (1998) [**14**]	533 1 MS-4 MS, USA	Chumbler measure [**13**]: Satisfaction subscale range 2(neg) to 14(pos); effectiveness subscale range 4(pos) to 28(neg)	1 MS and 2 MS satisfaction = 6.0; 1 MS and 2 MS effectiveness = 5.9; 3 MS and 4 MS satisfaction = 5.6; 3 MS and 4 MS Effectiveness = 5.8	High
De Biasio *et al*. (2016) [15]	404 1 MS-4 MS, USA	UCLA GAS [**35**]: Range 1(neg) to 5(pos)	1 MS = 3.8; 2 MS = 3.8, 3 MS = 3.7; 4 MS = 3.7	Low
Edwards and Aldous (1996) [16]	93 Doctors; 290 1 MS, 3 MS-5 MS, United Kingdom	ASD [**47**]: Range 1(pos) to 7(neg)	1 MS = 129.9; 3 MS = 126.7; 4 MS = 125.8; 5 MS = 124.8; Faculty: = 124.8	Low
Fields *et al.* (1992) [17]	127 4 MS, USA	ASD [**47**]: Range 32(pos) to 224(neg)	ASD = 130.5	Medium
Fitzgerald *et al.* (2003) [18]	171 1 MS, USA	UCLA GAS [**35**]: Range 1(neg) to 5(pos) Maxwell–Sullivan Scale [**30**]: Range 1(pos) to 5(neg)	UCLA GAS = 3.7; MSAS = 2.0	Medium
Hellbusch *et al.* (1995) [19]	200 Doctors, USA	Kogan's ATOP [**48**]: Range 34(pos) to 238(neg)	97.9	Medium
Hogan *et al*. (2014) [20]	173 Doctors, USA	UCLA GAS [**35**]: Range 1(neg) to 5(pos)	3.79	Medium
Hollar *et al*. (2011) [**21**]	116 1 MS, USA	UCLA GAS [**35**]: Range 14(neg) to 70(pos)	52.1	Medium
Holtzman *et al*. (1979) [22]	314 1 MS-4 MS, USA	ASD [**47**]: Range 32(pos) to 224(neg)	123.7	Medium
Holtzman *et al*. (1981) [23]	118 1 MS and 3 MS, USA	ASD [**47**]: Range 32(pos) to 224(neg)	1 MS = 119.1; 3 MS = 124.4	Low
Hughes *et al.* (2008) [24]	163 1 MS, UK	UCLA GAS [**35**]: Range 1(neg) to 5(pos)	3.7	Medium
Kishimoto *et al.* (2005) [25]	156 1 MS-3 MS; 55 doctors, USA	UCLA GAS [**35**]: Range 1(neg) to 5(pos)	1 MS = 3.9; 2 MS = 3.7; 3 MS = 3.6; PGY1^e^ = 3.6; PGY2 = 3.8; PGY3 = 3.7; Geriatric fellows = 4.1	Medium
Lee *et al*. (2005) [**26**]	177 Doctors, USA	UCLA GAS [**35**]: Range 1(neg) to 5(pos)	PGY1 = 3.5; PGY2 = 3.7; PGY3 = 3.6;	Medium
Leung *et al.* (2011) [**27**]	122 Doctors, Australia	Fraboni's Ageism Scale [59]: Range 29(pos) to 145(neg)	61.5	Medium
Linn and Zeppa (1988) [28]	179 3 MS, USA	Kogan's ATOP [**48**]: Range 1(neg) to 4(pos)	2.1	Medium
Lui and Wong (2009) [29]	54 Doctors, Singapore	Kogan's ATOP [**48**]: Range 34(neg) to 170(pos)	114.4	Low
Maxwell and Sullivan (1980) [**30**]	150 Doctors, USA	Maxwell–Sullivan Scale [**30**]: Range 1(pos) to 5(neg)	2.2	Low
Menz *et al*. (2003) [**31**]	81 3 MS and 4 MS, Australia	ASD [**47**]: Range 32(pos) to 224(neg) Chumbler measure Effectiveness [**13**]: Range 4(pos) to 28(neg)	ASD = 120.0; effectiveness scale = 18.6	Low
Muangpaisan *et al*. (2008) [32]	60 Doctors; 146 4 MS, Thailand	UCLA GAS [**35**]: Range 16(pos) to 80(neg)	4 MS = 41.8; Doctors = 40.7	Low
Perrotta *et al.* (1981) [33]	127 1 MS, USA	Kogan's ATOP [**48**]: Range 34(pos) to 170(neg)	81.0	Medium
Reuben *et al.* (1995) [**34**]	554 1 MS, USA	ASD [**47**]; Maxwell–Sullivan Scale [**30**]: Range 32(pos) to 224(neg)	128.4	High
Reuben *et al.* (1998) [**35**]	142 doctors, USA	UCLA GAS [**35**]: Range 1(neg) to 5(pos)	PGY1 = 3.4; PGY2 = 3.6, PGY3 = 3.8; Fellows = 4.2; Faculty = 4.2	Low
Richter and Buck (1990) [**36**]	85 doctors, USA	Maxwell–Sullivan Scale [**30**]: Range 28(neg) to 140(pos)	PGY1 = 102.0; PGY2 = 105.3, PGY3 = 105.6; Faculty = 104.3	Low
Ruiz *et al.* (2015) [**37**]	103 1 MS-4 MS, USA	Fraboni's Ageism Scale [59]: Range 29(pos) to 145(neg)	67	Low
Sainsbury *et al*. (1994) [38]	68 Doctors, New Zealand	ASD [**47**]: Range 32(pos) to 224(neg)	115.8	Low
Shahidi and Devlen (1993) [39]	84 2 MS, UK	ASD [**47**]: Range 32(pos) to 224(neg)	89.6	Low
Thorson and Powell (1991) [**40**]	277 1 MS, USA	Kogan's ATOP [**48**]: Range 1(pos) to 7(neg)	102.6	Low
Voogt *et al.* (2008) [**41**]	231 1 MS, USA	UCLA GAS [**35**]: Range 1(neg) to 6(pos)	4.5	Medium
Wilderom *et al.* (1990) [**42**]	663 1 MS, USA	Kogan's ATOP [**48**]: Range 34(pos) to 170(neg)	81.9	Medium
Yang *et al.* (2013) [**43**]	270 Doctors, China	ASD [**47**]: Range 1(pos) to 5(neg); Palmore's Bias Score [**50**]: Range −100(neg) to 100(pos)	ASD = 2.6; FAQ1 Bias score = −17.3	High
Zverev (2015) [44]	154 1 MS-5 MS, Malawi	Kogan's ATOP [**48**]: Range 34(neg) to 204(pos)	129.1	Medium

^a^More detailed information about the main threats to quality are included in Supplementary Data (Appendix C).

^b^1 MS–5 MS denotes the year the students included in the study were in medical school from Year 1 Medical Student (i.e. 1 MS) to Year 5 Medical Student (i.e. 5 MS).

^c^ASD: Aging semantic differential.

^d^GAS: Geriatrics attitude scale, ± neg denotes negative attitudes and pos denotes positive attitudes.

^e^PGY1–PGY3 denotes year in postgraduate medical training from Postgraduate Year 1 (PGY1) to Postgraduate Year 3 (PGY3), +ATOP: attitudes toward old people scale

### Design and quality of studies

Typically, studies failed to provide details of a power calculation to justify the size of the sample. Eight studies used very small samples of <100 participants [**8**, 16, 29, **31**, 32, **36**, 38, 39], which can be expected to have weak power to detect relationships [45] and thus indicated low quality. Using the STROBE checklist to evaluate quality, the main weaknesses across studies related to response rates, sample size and the instrument employed. Eight studies also had unreported or low response rates (<40%) which was considered an indicator of low quality [15, 23, **30**, **31**, **35**, **37**, 39, **40**]. Five studies were considered high quality as they had reported over 65% response rates, had over 250 participants (which has been identified as a point at which correlations stabilise [46]) and used a measure which had reliability or validity evidence based on samples with healthcare professionals [**10**, **13**, **14**, **34**, **43**]. Overall judgements of low, medium and high quality studies are also included in Table 1. Further results from the quality assessment exercise and characteristics of included studies are available in Table S1 and as part of the Supplementary Data available online (Appendix C).

#### Measures employed

Studies commonly did not report the psychometric properties of the chosen measure of attitudes where a previously established scale was used, or failed to establish these properties for locally developed *de novo* measures. Eleven studies used the UCLA Geriatric Attitude Scale [**35**] (GAS) which is a questionnaire specifically designed to measure attitudes toward older patients (rather than older people in general) and has validity and reliability evidence based on studies with healthcare professionals. A further 11 used the Aging Semantic Differential [**47**], a well-established general purpose tool for measuring attitudes towards older people. Nine studies employed Kogan's Attitude toward Old People (KAOP) scale [**48**], but the use of this measure to ascertain attitudes is questionable. It is a 55-year old questionnaire consisting of items relating to stereotypes (e.g. ‘most old people are pretty much alike’), without the corresponding affective information regarding whether the respondent thinks this is a positive or negative attribute (i.e. their attitude) [49]. Furthermore, a number of stereotypes are likely to be irrelevant to medical students’ and doctors’ attitudes toward older patients (e.g. ‘most old people tend to let their homes become shabby and unattractive’ p. 46) [**48**].

### Variables related to attitudes towards older patients

Relationships between variables of interest and attitudes toward older patients across the 37 studies are demonstrated in Table 2.

**Table 2.** Variables related to attitudes toward older patients reported by included studies

Variable	Significant positive relationship with attitudes	Significant negative relationship with attitudes	No significant relationship with attitudes reported	Total studies
**Demographics**				
Gender: female	[13^a^-H^b^]; [14-H]; [15-L]; [18-M]; [21-M]; [27-M]; [31-L]; [34-H]; [37-L]; [43-H]		[8-L]; [9-M]; [10-H]; [11-M]; [12-M]; [16-L]; [17-M]; [18-M]; [19-M]; [22-M]; [24-M]; [32-L]; [35-L]; [39-L]; [41-M]; [42-M]; [43-H]; [44-M]	28
Increasing age	[10-H]; [15-L]; [27-M]; [34-H]	[17-M]; [19-M]	[8-L]; [9-M]; [12-M]; [22-M]; [23-L]; [24-M]; [29-L]; [31-L]; [32-L]; [35-L]; [39-L]; [41-M]; [42-M]; [43-H]	20
Marital status			[29-L]; [34-H]	2
Ethnicity: White	[13-H]		[9-M]; [11-M]; [12-M]; [14-H]; [15-L]; [18-M]; [24-M]; [35-L]; [37-L]; [41-M]	11
Ethnicity: Asian-American		[26-M]; [34-H]		2
Nationality/country of birth			[27-M]; [29-L]	2
Socioeconomic background			[11-M]; [13-H]	2
Having doctor parent			[11-M]	1
Languages spoken			[27-M]	1
**Education and training**				
Years in medical school	[25-M]	[15-L]	[11-M]; [13-H]; [16-L]; [22-M]; [23-L]; [37-L]; [44-M]	9
Med school attended	[9-M]	[29-L]		2
Increasing years of practice/seniority	[27-M]; [30-L]; [35-L]	[19-M]; [25-M]	[8-L]; [20-M]; [29-L]; [36-L]; [38-L]; [43-H]	11
Prior geriatrics course	[36-L]		[8-L]; [10-H]; [14-H]; [17-M]; [19-M]; [34-H]; [42-M]	8
Prior science-related course			[15-L];	1
Faculty attitude scores	[36-L]			1
Completed geriatrics rotation			[38-L]	1
**Exposure to older people**				
Knowledge of older people	[16-L]; [23-L]; [28-M]; [31-L]; [33-M]; [34-H]; [39-L]; [43-H]		[8-L]; [17-M]; [18-M]	11
Contact with older people	[26-M]; [27-M]; [32-L]; [41-M]; [42-M]		[10-H]; [17-M]; [31-L]; [33-M]; [36-L]	10
Older people care experience	[41-M]; [42-M]		[10-H]; [12-M]; [17-M]; [18-M]; [24-M]; [34-H]	8
Age of parents			[34-H]; [36-L]	2
**Personality and cognitive**				
Cognitive ability			[9-M]	1
Orientation to authority			[9-M]	1
Level of intrinsic motivation	[9-M]; [13-H]; [14-H]; [37-L]		[31-L]	5
Level of extrinsic motivation		[9-M]; [13-H];		2
Dominance personality trait		[40-L]		1
Social competence			[15-L]; [42-M]	2
**Job and career**				
Interest in working with older people/geriatrics	[12-M]; [18-M]; [24-M]; [26-M]; [27-M]; [28-M]; [35-L]; [37-L]; [41-M]; [42-M]		[33-M]	11
Clinical contact with older patients	[14-H]; [26-M]		[19-M]	3
Interest in family medicine	[22-M]; [28-M]		[9-M]; [13-H]; [14-H]; [17-M]; [19-M]; [35-L]; [42-M]	9
City versus rural location preference			[9-M]	1
Private vs public sector preference			[31-L]	1
Preference for older patients	[42-M]		[18-M]	2

^a^Studies are identified by their citation number in the reference list. The full reference list is included in Supplementary Data (Appendix D). ^b^H/M/L denotes High/Medium/Low quality ratings as indicated in Table 1. More detailed information about the main features related to quality is included in Supplementary Data (Appendix C).

#### Demographic factors

The most commonly investigated demographic factors were gender, age, race or ethnicity. A total of 28 studies examined the link between gender and attitudes, and 18 reported no evidence of a relationship [**8**–12, 16–19, 22, 24, 32, **35**, 39, **41**–44]. All 10 studies which found a relationship reported that female respondents had more positive attitudes than male respondents [**13**–15, 18, **21**, **27**, **31**, **34**, **37**, **43**]. All five high quality studies in this review investigated the relationship between gender and attitudes with four of these studies reporting more positive attitude scores for females.

The majority of studies investigating relationships between race and attitudes, and age and attitudes reported that there was no significant relationship between these variables. Where a relationship between attitudes and age was reported, studies often did not recognise the likelihood of multi-collinear associations between age and other variables (e.g. years in medical school or years of practice) and may therefore have violated statistical test assumptions.

#### Education and training

On the whole, variables related to stage of education or career, previous education in, and clinical experience of geriatric medicine did not appear to be related to the attitudes of medical students and doctors toward older patients. Richter and Buck [**36**] reported that doctors’ attitude scores were significantly associated with the attitude scores of teaching faculty members at their residency (training) programmes.

#### Exposure to older people

Ten studies investigated the relationship between respondents’ personal contact with older people (including older relatives) and attitude scores, and the results were mixed. Five studies reported no significant relationship between variables [**10**, 17, **31**, 33, **36**]; these studies all posed questions pertaining to the frequency of contact with older people. The remaining five studies did report associations [**26**, **27**, 32, **41**, **42**] and these all posed questions relating to the quality of the relationship with the older person. Therefore, it appears likely that the quality of contact or relationships with older people may be related to attitude scores but the frequency of contact may not. Respondents’ knowledge about older people and the ageing process was investigated in 11 studies, with eight studies reporting that higher knowledge scores were associated with more positive attitudes [16, 23, 28, **31**, 33, **34**, 39, **43**], with three studies reporting no evidence of a relationship [**8**, 17, 18]. All eight studies demonstrating such a relationship used Palmore's Facts on Aging Quizzes (FAQs) [**50**, **51**], which has been demonstrated to conflate knowledge and attitudes to the extent that they cannot be analysed separately [**50**].

#### Personality and cognitive factors

Five studies investigated intrinsic motivation for entering medicine (such as becoming a doctor to help others) and four out of five studies found those reporting high intrinsic motivation were more likely to report more positive attitude scores [**9**, **13**, **14**, 29]. When low quality studies were removed, three out of three studies reported a relationship between intrinsic motivation and attitudes. Two studies measured extrinsic motivation for entering medicine (such as future earning potential and job security) with both studies reporting that negative attitudes towards older patients were associated with higher levels of extrinsic motivation [**9**, **13**]. Personality factors that did not provide evidence of any relationship with attitude scores included respondents’ orientation to authority [**9**], cognitive ability [**9**], and self-reported social competence [15, **42**].

#### Job and career factors

Ten of 11 studies found that respondents reporting greater levels of interest in geriatric medicine or preference to work with older patients had more positive attitudes toward older patients [12, 18, 24, **26**–28, **35**, **37**, **41**, **42**]. This relationship was still evident when low quality studies were removed from the analysis.

## Discussion

### Key findings

This review of 37 studies examined the relationships between attitudes towards older patients and a range of demographic, educational and professional-related variables. The findings indicated that the quality of previous relationships with older people was linked to attitudes, with good quality relationships with older people (such as grandparents and family friends) related to positive attitudes. This finding supports results from intervention studies that have reported mentorship programmes between community-dwelling healthy older people and medical students to produce positive attitude changes [**4**]. The results from this review also linked a preference for working with older patients with positive attitudes to older patients. Given that the majority of studies included medical students, this finding suggests that measuring attitudes towards older patients, even in the early stages of medical school, might allow for the better identification of those who are suited to, and may enjoy, working with older patients. Another key finding was the link between intrinsic motivation and attitudes towards older patients. The identification of intrinsic motivation for entering medicine may present an opportunity to help screen for medical students who are more inclined to work with the increasing number of older patients presenting in healthcare.

The findings concerning the relationship between attitudes and knowledge about ageing are consistent with those previously reported for nurses [**5**]. All studies reporting this relationship in the present review and in the systematic review of the nursing literature [**5**] used Palmore's FAQs [**50**, **51**] to measure knowledge. The FAQs [**50**, **51**] were intentionally designed by Palmore to have a secondary purpose as an indirect measure of attitudes in addition to their primary purpose as a knowledge measure [**50**]. Only one out of the eight studies, reporting a relationship between attitudes and knowledge, acknowledged in its discussion that the knowledge measure is an indirect attitude measure which may account for the relationships reported [**43**]. Future research should seek to employ a purpose-built knowledge measure which is not also designed to measure attitudes, and we would additionally recommend the use of a knowledge measure relevant to older patients’ medical care. For example, Lee *et al.* [52] have designed knowledge measures specifically for testing clinically relevant knowledge in medical students or doctors, which have demonstrated reliability and validity evidence. Higher scores on this measure have shown correlations with enhanced clinical skill levels [52]. The wider literature supports the view that knowledge and attitudes are distinct, which is supported by the finding that geriatric interventions with a knowledge-building focus have been unsuccessful at changing attitudes [**4**].

The finding that geriatric education and training was not reported as being associated with positive attitudes towards older patients, calls into question current assumptions about the effect of early exposure and later interest in geriatric medicine. However, one study in this review found that doctors’ attitudes correlated with those of teaching faculty on their residency programmes, which reinforces the importance of professional socialisation and the ‘hidden curriculum’ in shaping attitudes. Potentially, interventions to improve medical students’ and doctors’ attitudes may be unsuccessful if not reinforced or supported by exposure to positive attitudes amongst teaching faculty members. Future attitudinal interventions may benefit from vertically extending the targets of interventions in medical schools to include faculty.

The findings related to gender (where four out of five of the high quality studies reported more positive attitude scores in females compared to males) warrants further enquiry. It is possible that higher scores reported by females represent social desirability or impression management (being able to manage the impression they leave on others): female medical students report higher social desirability/impression management scores than males [53]. Alternatively, the positive attitude scores may reflect more socially responsible attitudes in female medics [54], as female medical students’ ethical attitudes have been found to remain stable and more positive toward those in underserved patient populations, compared to their male peers whose attitudes toward underserved patients declined throughout medical school [54]. Another possible explanation for gender differences may relate to tolerating clinical uncertainty. Male medical students report higher aversion to clinical uncertainty than females, which has been associated with a more negative attributional style for dealing with geriatric patients, and a greater likelihood of avoiding complex specialties such as family and internal medicine [**55**]. Differences between male and female doctors in tolerating uncertainty (which includes dealing with risk and complexity) could have implications for older patient care outcomes as well as attitudes towards this group and the decision to work with them. For example, intolerance to uncertainty has been linked to a failure to follow evidence-based guidelines [56], increased propensity to order diagnostic tests and performing unnecessary investigations on patients to reduce uncertainty [57]. Recent evidence highlights the need to further understand how gender-related factors may influence older patient care: Tsugawa *et al.* report reduced mortality and readmissions for older patients under the care of female internists. They hypothesised this was related, in part, to differences in how male and female doctors deal with complex problems [58].

### Strengths and limitations of this review

The strengths of this review include the use of search terms which were broad and inclusive; searching 10 online databases and snowballing references lists of included studies; the addition of articles from over four decades; and the assessment of quality using standardised tools. We sought to provide as comprehensive an overview as possible. We thus assessed and presented data from lower quality studies in addition to higher quality studies. Where exclusion criteria were applied, this was done not on the basis of methodological quality but to ensure that all articles included in this review provided evidence that they measured what they purported to measure. This was necessary to ensure that meaningful conclusions were drawn. We believe that this clear rationale for the applied exclusion criteria represents a strength. That this review is limited to research in English may mean that insights from papers written in other languages have been missed. Attitudes and behaviours are complex constructs and very detailed interpretative translation would have been required to make sense of papers published in other languages. The use of a single reviewer to conduct quality assessments will have increased the consistency with which assessments were conducted, but increased the risk of systematic bias. Another important limitation is the decision to exclude data from the qualitative and grey literature, which could have increased the breadth of insights generated. For instance, not all educational scholarship is published in the peer-reviewed literature and cited in healthcare-focussed bibliographic databases.

## Conclusion

This review identifies three factors, which indicate an association with attitudes towards older patients: motivation for entering medicine; preference to work with older patients; and the quality of previous relationships with older people. Additionally, the two factors of gender and knowledge of ageing represent an opportunity for more robust enquiry to determine if a relationship exists between these and attitudes in such a way that might inform selection to the medical profession, or specific training programmes within medicine, at a time when high quality engagement between healthcare professionals and older patients is of the essence.

**Key points**

- Three factors were related to attitudes towards older patients.
- Related factors included motivation, preference for gerontology work and quality of past relationships with older people.
- Factors associated with positive attitudes may help the early identification of those well suited to working with older patients.
- The link between knowledge and attitudes may relate to the use of Palmore's Facts on Aging quizzes to test knowledge.
- Future research could further explore whether females report more positive attitudes towards older patients.

## Supplementary data

Supplementary data mentioned in the text are available to subscribers in *Age and Ageing* online.

## Supplementary Material

Supplementary Data

## References

[afx058C1a] PLEASE NOTE: The very long list of references supporting this review has meant that only the most important are listed here and are represented by bold type throughout the text. The full list of references is available on the journal website http://www.ageing.oxfordjournals.org/ as Appendix D.

[1] Oliver D, Burns E Geriatric medicine and geriatricians in the UK. How they relate to acute and general internal medicine and what the future might hold? Future Hosp J 2016; 3: 49–54. 10.7861/futurehosp.3-1-49 PMC6465863 31098179

[2] Oakley R, Pattinson J, Goldberg S et al Equipping tomorrow's doctors for the patients of today. Age Ageing 2014; 43: 442–7. 24958744 10.1093/ageing/afu077

[3] Tullo ES, Spencer J, Allan L Systematic review: helping the young to understand the old. Teaching interventions in geriatrics to improve the knowledge, skills, and attitudes of undergraduate medical students. J Am Geriatr Soc 2010; 58: 1987–93. 20840458 10.1111/j.1532-5415.2010.03072.x

[4] Samra R, Griffiths A, Cox T et al Changes in medical student and doctor attitudes toward older adults after an intervention: a systematic review. J Am Geriatr Soc 2013; 61: 1188–96. 23750821 10.1111/jgs.12312 PMC3808566

[5] Liu Y, Norman IJ, While AE Nurses’ attitudes towards older people: a systematic review. Int J Nurs Stud 2013; 50: 1271–82. 23265870 10.1016/j.ijnurstu.2012.11.021

[7] von Elm E, Altman DG, Egger M et al The Strengthening the Reporting of Observational Studies in Epidemiology (STROBE) statement: guidelines for reporting observational studies. Lancet 2007; 370: 1453–7. 18064739 10.1016/S0140-6736(07)61602-X

[8] Beall C, Baumhover LA, Simpson JA et al Teaching geriatrics medicine: resident's perceptions of barriers and stereotypes. Gerontol Geriatr Educ 1991; 11: 85–96.

[9] Belgrave LL, Lavin B, Breslau N et al Stereotyping of the aged by medical students. Gerontol Geriatr Educ 1982; 3: 37–44.

[10] Cammer Paris BE, Gold G, Taylor B et al First year medical student attitudes toward the elderly: a comparison of years 1986, 1991 and 1994. Gerontol Geriatr Educ 1997; 18: 13–22.

[13] Chumbler NR, Robbins JM, Poplawski ME Rewards of entering pediatric medicine and attitudes toward older adults. J Am Podiatr Med Assoc 1996; 86: 288–94. 8699353 10.7547/87507315-86-6-288

[14] Chumbler NR, Ford TE The orientation of health professional students towards the care of older adults: the case of podiatry. Health 1998; 2: 259–81.

[21] Hollar D, Roberts E, Busby-Whitehead J COCOA: a new validated instrument to assess medical students’ attitudes towards older adults. Educ Gerontol 2011; 37: 193–209.

[26] Lee M, Reuben DB, Ferrell BA Multidimensional attitudes of medical residents and geriatrics fellows toward older people. J Am Geriatr Soc 2005; 53: 489–94. 15743295 10.1111/j.1532-5415.2005.53170.x

[27] Leung S, LoGiudice D, Schwarz J et al Hospital doctors’ attitudes towards older people. Int Med J 2011; 41: 308–14. 10.1111/j.1445-5994.2009.02140.x 20002850

[28] Linn BS, Zeppa R Predicting third year medical students’ attitudes toward the elderly and treating the old. Gerontol Geriatr Educ 1987; 7: 167–75. 3453341 10.1300/j021v07n03_14

[30] Maxwell AJ, Sullivan N Attitudes toward the geriatric patient among family practice residents. J Am Geriatr Soc 1980; 28: 341–5. 7400502 10.1111/j.1532-5415.1980.tb01095.x

[31] Menz HB, Stewart FA, Oates MJ Knowledge of aging and attitudes toward older people—a survey of Australian podiatric medical students. J Am Podiatr Med Assoc 2003; 93: 11–7. 12533550 10.7547/87507315-93-1-11

[34] Reuben DB, Fullerton JT, Tschann JM et al Attitudes of beginning medical students toward older persons: a five-campus study. The University of California Academic Geriatric Resource Program Student Survey Research Group. J Am Geriatr Soc 1995; 43: 1430–6. 7490398 10.1111/j.1532-5415.1995.tb06626.x

[35] Reuben DB, Lee M, Davis JW et al Development and validation of a geriatrics attitudes scale for primary care residents. J Am Geriatr Soc 1998; 46: 1425–30. 9809767 10.1111/j.1532-5415.1998.tb06012.x

[36] Richter RC, Buck EL Family practice residents and the elderly: fostering positive attitudes. Fam Med 1990; 22: 388–91. 2227176

[37] Ruiz JG, Andrade AD, Anam R et al Group-based differences in anti-aging bias among medical students. Gerontol Geriatr Educ 2015; 36: 58–78. 25288486 10.1080/02701960.2014.966904

[40] Thorson JA, Powell FC Medical students’ attitudes towards ageing and death: a cross-sequential study. Med Educ 1991; 25: 32–7. 1997826 10.1111/j.1365-2923.1991.tb00023.x

[41] Voogt SJ, Mickus M, Santiago O et al Attitudes, experiences, and interest in geriatrics of first-year allopathic and osteopathic medical students. J Am Geriatr Soc 2008; 56: 339–44. 18086123 10.1111/j.1532-5415.2007.01541.x

[42] Wilderom CP, Press EG, Perkins DV et al Correlates of entering medical students’ attitudes toward geriatrics. Educ Gerontol 1990; 16: 429–46.

[43] Yang Y, Xiao LD, Ullah S et al General practitioners’ knowledge of ageing and attitudes towards older people in China. Australas J Ageing 2015; 34: 82–7. 24118793 10.1111/ajag.12105

[47] Rosencranz HA, McNevin TE A factor analysis of attitudes toward the aged. Gerontologist 1969; 9: 55–9. 5769675 10.1093/geront/9.1.55

[48] Kogan N Attitudes toward old people: the development of a scale and an examination of correlates. J Abnorm Soc Psychol 1961; 62: 44–54. 13757539 10.1037/h0048053

[50] Palmore E Facts on aging: a short quiz. Gerontologist 1977; 17: 315–20. 892527 10.1093/geront/17.4.315

[51] Palmore E The facts on aging quiz: Part two. Gerontologist 1981; 21: 431–7.

[55] Merrill JM, Camacho Z, Laux LF et al Uncertainties and ambiguities: measuring how medical students cope. Med Educ 1994; 28: 316–22. 7862004 10.1111/j.1365-2923.1994.tb02719.x

