# Supplementary material for: Factors related to medical students’ and doctors’ attitudes towards older patients: a systematic review
Source: Age Ageing. 2017 May 2;46(6):911–9. doi: 10.1093/ageing/afx058 (PMC5860378; doi:10.1093/ageing/afx058)
Supplement: Supplementary Data [file afw058aa-17-0020.r1supplementarydataappendicesvs2.docx]

**Supplementary Appendix A**

**Systematic Review Protocol**

**Title:**

Factors related to medical students’ and doctors’ attitudes towards older patients: A systematic review

**Authors:** Rajvinder Samra, Tom Cox, Adam L Gordon, Simon Conroy, Mathijs Lucassen & Amanda Griffiths

**Review question(s)**

What variables are related to medical students and doctors attitudes toward older patients?

**Condition or domain being studied**

Variables related to medical students’ and doctors’ attitudes toward older patients as measured by self-report studies. All variables considered of interest were investigated for a possible relationship with attitudes towards older patients. Studies presented correlational analyses or difference testing between attitudes towards older patients and other variables. Significant and non-significant relationship data were extracted.

**Search strategy**

The search strategy was developed by three members of the core research team (RS, AG & TC), an independent researcher and one subject librarian were consulted to maximise retrieval of relevant studies.

The search terms were [in title or abstract]: (Physician [indexed term]; doctor; physician; clinician; hospitalist; internist; surgeon; general practitioner; family practitioner; medical student; medical professional; medical provider; medical practitioner) AND (Aged [Indexed term]; Old* person; old* patient; old* adult; elder*; frail; aging; ageing; aged patient; aged person; geriatric patient; geriatric person; old age; senior citizen; senior adult; senior person; senior patient) AND (Attitude [indexed term]; Attitud*; belief; ageis; agis*; discriminat*; prejudic*; stereotyp*; stigma; labeling; labelling; age bias).

In order to minimise the number articles that were missed due the reliance on online databases additional techniques were employed including:

• Searching the reference lists of included studies;

• Citation tracking of the included studies

• Internet searches (e.g. google scholar)

• Personal knowledge and academic contacts to identify any missed studies.

For further details of the search across the ten databases, please contact the review team via the contact details supplied at the end of this protocol.

**Search**

- The following databases were searched:
- ABI/Inform (from 1988)
- Applied Social Sciences Index & Abstracts (from 1987)
- British Nursing Index (from 1994)
- Cumulative Index to Nursing & Allied Health (from 1981)
- Informa Health (from 1997)
- Medline (from 1965)
- PsycInfo (from 1887)
- Science Direct (from 1995)
- Scopus (from 1966)
- Web of Science (from 1900)

**Inclusion criteria:**

Studies were included if:

- Participants were medical students and/or medical practitioners (doctors);
- They quantitatively measured attitudes toward adults aged 65 and older using a questionnaire design;
- They investigated variables associated with attitudes to older adults/patients;
- They provided overall mean scores for attitude measures;
- They were available in English in a peer-reviewed journal.

**Exclusion criteria:**

Studies were excluded if they:

- Did not report the measure used or failed to report the items employed to measure attitudes;
- Did not report results of inferential tests;
- Did not present attitude data from medical professionals (medical students and doctors) separately from other groups of individuals;
- Evaluated an intervention without correlational analyses (e.g. providing pre- and post-test attitude scores only);
- Duplicated findings from another study already included in the review.

**Comparator(s)/ control**

Not applicable

**Participants/Population of interest**

Respondents must be medical students or medical doctors.

**Outcome(s)**

Variables that correlate or demonstrate a relationship with scores on attitudes towards older patients/adults measure.

**Types of study to be included:**

Quantitative, English language, original data. No conference abstracts, letters, opinion pieces, qualitative studies, or narrative articles, and no reviews.

**Quality assessment:**

A data extraction form by the Long et al. [1] was modified to meet the requirements of this review. Quality was assessed using the Evaluation Tool for Quantitative Research Studies [1] and using the criteria identified in the Strengthening the Reporting of Observational Studies (STROBE) statement [2].

References:

1. Long A, Godfrey M, Randall T, et al. Developing evidence based social care policy and practice. Part 3: feasibility of undertaking systematic reviews in social care. University of Leeds (Nuffield Institute for Health) and University of Salford (Health Care Practice R&D Unit) 2002.

2. von Elm, E., et al., The Strengthening the Reporting of Observational Studies in Epidemiology (STROBE) statement: guidelines for reporting observational studies. Lancet, 2007. 370(9596): p. 1453-1457.

**Data extraction:**

Data extracted comprised: bibliographic details of the study (author names and date published), setting (location of study), participant characteristics (age range, medical school year group, or doctor grade), study design (study type, number of groups), time of assessment (year), sample selection (size of source group, selection method, sample size justification, response rates), study method (control of confounders), study instruments (instruments used, outcome measurement criteria, validity and reliability reports, attitude scores), data analysis methods (suitability of statistical techniques), variables investigated for a relationship with attitude scores and results (significant associations or differences, non-significant results), and process issues (reported problems in data collection).

**Analysis of subgroups or subsets**

The majority of the studies focused on medical students and so there was insufficient comparative data on the doctor-only group to warrant a separate subgroup.

**Dissemination plans**

The review will be published in a peer reviewed journal

**Contact details for further information:**

Rajvinder Samra, H20, Horlock Building, The Open University, Walton Hall, Milton Keynes, MK7 6AA, UK; Tel: +44 1908 666516; E-mail: r.samra@mail.com

**Review team**

Rajvinder Samra, Tom Cox, Mathijs Lucassen & Amanda Griffiths

**Start date**

15 March 2011

**Completion date**

10 February 2017

**Funding sources/sponsors**

This research was supported by an Economic and Social Research Council (United Kingdom) studentship to RS (Grant Reference: ES/H014659/1)

**Conflicts of interest**

None known

**Language**

English

**Country**

UK

**Subject index terms**

Attitude; Medical students; doctors; older patients; older adults

**Stage of review**

Completed and submitted for publication

**Date of protocol**

10 February 2017

**Stage of review at time of this protocol Started Completed**

Preliminary searches Yes Yes

Piloting of the study selection process Yes Yes

Formal screening of search results against eligibility criteria Yes Yes

Data extraction Yes Yes

Risk of bias (quality) assessment Yes Yes

Data analysis Yes Yes

**Supplementary Appendix B**

**Search strategy (Medline)**

**Step Query**

S1 ((MM "Physician-Patient Relations") OR (MH "Physicians+") OR (MM "Students, Medical"))

S2 TI ((doctor OR physician OR clinician OR hospitalist OR internist OR surgeon OR "general practitioner" OR "family practitioner") OR (medical W1 (student OR intern OR professional OR provider OR practitioner))) OR AB((doctor OR physician OR clinician OR hospitalist OR internist OR surgeon OR "general practitioner" OR "family practitioner") OR (medical W1 (student OR intern OR professional OR provider OR practitioner)))

S3 S1 OR S2

S4 (MM "Aged")

S5 TI (("old* person" OR "old* people" OR "old* patient?" OR "old* adult?" OR "elder*" OR "frail" OR "aging" OR "ageing" OR "aged patient" OR "aged person" OR "geriatric patient?" OR "geriatric person" OR "old age" OR "senior citizen?" OR "senior adult?" OR "senior person" OR "senior patient?"))

S6 S4 OR S5

S7 (MM "Attitude of Health Personnel")

S8 TI ( ("Attitud*" OR "belief" OR "ageis*" OR "agis*" OR "discriminat*" OR "prejudic*" OR "stereotyp*" OR "stigma" OR "age bias") ) OR AB ( ("Attitud*" OR "belief" OR "ageis*" OR "agis*" OR "discriminat*" OR "prejudic*" OR "stereotyp*" OR "stigma" OR "age bias") )

S9 S7 OR S8

S10 (MM "Ageism")

S11 TI("ageis*" OR "agis*") OR AB("ageis*" OR "agis*")

S12 S10 OR S11

S13 S3 AND S12

S14 S3 AND S6 AND S9

S15 S13 OR S14

S16 ((MM "Physician-Patient Relations") OR (MH "Physicians+") OR (MM "Students, Medical"))

S17 TI ((doctor OR physician OR clinician OR hospitalist OR internist OR surgeon OR "general practitioner" OR "family practitioner") OR (medical student OR medical intern OR medical professional OR medical provider OR medical practitioner))) OR AB((doctor OR physician OR clinician OR hospitalist OR internist OR surgeon OR "general practitioner" OR "family practitioner") OR (medical student OR medical intern OR medical professional OR medical provider OR medical practitioner)))

S18 S16 OR S17

S19 (MM "Aged")

S20 TI (("old* person*" OR "old* people" OR "old* patient*" OR "old* adult*" OR "elder*" OR "frail" OR "aging" OR "ageing" OR "the aged" OR "aged patient" OR "aged person" OR "geriatric patient?" OR "geriatric person" OR "old age" OR "senior citizen?" OR "senior adult?" OR "senior person" OR "senior patient?"))

S21 S19 OR S20

S22 (MM "Attitude of Health Personnel")

S23 TI ( ("Attitud*" OR "belief" OR "ageis*" OR "agis*" OR "discriminat*" OR "prejudic*" OR "stereotyp*" OR "stigma" OR "age bias") ) OR AB ( ("Attitud*" OR "belief" OR "ageis*" OR "agis*" OR "discriminat*" OR "prejudic*" OR "stereotyp*" OR "stigma" OR "age bias") )

S24 S22 OR S23

S25 (MM "Ageism")

S26 TI("ageis*" OR "agis*") OR AB("ageis*" OR "agis*")

S27 S25 OR S26

S28 S18 AND S27

S29 S18 AND S21 AND S24

S30 S28 OR S29

**Supplementary Appendix C:**

Supplementary Table S1 *Characteristics of included studies*

| Authors | Sample, setting, & response rate (RR) | Attitude assessment | Significant relationship reported with attitude score | No significant relationship reported with attitude score | Main threats to quality using STROBE checklist | Conclusions |
| --- | --- | --- | --- | --- | --- | --- |
| Beall, Baumhover, Simpson, & Pieroni (1991) [8] | 30 doctors, United States  Response rate 47% | Kogan’s Attitudes Toward Old People Scale [48] | n.r.‡ | (1) Gender  (2) age  (3) undergraduate major  (4) year of residency  (5) knowledge score | *Statistical:* Small group size; no power estimation or justification of sample size; statistical tests unclear. | Variables studied showed no relationship with attitude scores. |
| Belgrave, Lavin, Breslau, & Haug (1982) [9] | 120 medical students  (1MS)†,  United States  Response rate 80% | Palmore’s Bias score [50] | (1) Attending research-oriented school (r=-.35)  (2) seeing medicine as an ‘exciting job’ (r=.19)  (3) ‘helping others’ as reason for medical career choice (r=.19) | (1) Gender  (2) race  (3) age  (4) orientation to authority  (5) Medical College Admission Test (MCAT) verbal  (6) MCAT quantitative score  (7) preferred specialty  (8) preferred future location choice | *Measurement:* no justification of major change to measure; used knowledge questionnaire to measure attitudes.  *Statistical*: Significance levels chosen are unclear; no power estimation. | Attitude scores were more positive for those attending a community-focused medical school as opposed to a research focused school, and those who reported entering medicine to help others. Attitudes scores were more negative in those who reported entering medicine because it was an exciting job. |
| Cammer-Paris, et al. (1997) [10] | 330 medical students:  (1MS) from three cohorts (1986, 1991 & 1994),  United States  Response rate 90% | Aging Semantic Differential [47] | (1) Age (r=-12) | (1) Gender  (2) exposure to a nursing home  (3) previous undergraduate course in geriatrics.  (4) amount of contact with older persons | *Bias:* Mean attitude score difference between cohorts - no explanation given.  *Measurement:* Different measures used on the different cohorts – no justification. | Older medical students had more positive attitude scores. |
| Cheong, Wong, & Koh (2009) [11] | 342 medical students  (1MS & 3MS),  Singapore  Response rate 95% | Kogan’s Attitudes Toward Old People Scale [48] | n.r. | (1) Gender  (2) year of course  (3) ethnicity  (4) household income  (5) having a doctor as a parent | *Statistical:* Unclear justification of statistical testing choices; no power estimation. | Variables studied showed no relationship with attitude scores. |
| Chua, Chay, Merchant, & Soiza (2008) [12] | 244 medical students  (1MS),  Singapore  Response rate 98% | UCLA Geriatric Attitudes Scale [35] | (1) Willingness to consider career in geriatric medicine (r=.48) | (1) Gender  (2) age  (3) ethnicity  (4) previous experience caring for older people | *Bias:* Students’ data was identifiable – possible socially desirable responding  *Statistical:* No power estimation. | Medical students who were more willing to consider a geriatric medicine career had more positive attitude scores. |
| Chumbler, Robbins, & Poplawski (1996) [13] | 481 medical students  (2MS & 3MS) from a randomly selected national sample,  United States  Response rate 70% | Locally developed scale: Expected satisfaction & effectivene-ss in treating older adults scale. | (1) Gender (beta=.11)  (2) ethnicity (beta=.10)  (3) level of intrinsic motivation (r=.56)  (4) level of extrinsic motivation (r=.55) | (1) Year of course  (2) future residency choice  (3) socioeconomic background index | *Measurement:* Low reliability of measure.  *Participants:* Year group numbers not provided.  *Statistical:* No power estimation. | Attitude scores were more positive in female respondents, white respondents, and those who reported higher levels of intrinsic motivation as a reason for entering medicine. Negative attitude scores were more likely in those reporting greater levels of extrinsic motivation for entering medicine. |
| Chumbler & Ford (1998) [14] | 533 medical students  (1MS-4MS) from a randomly selected national sample,  United States  Response rate 76% | Chumbler et al. attitude measure [13] | 1MS & 2MS:  (1) gender (beta=-.13)  (2) level of intrinsic motivation (beta range for two subscales=.08 to .37)  3MS & 4MS:  (1) gender (beta=0.20)  (2) level of intrinsic motivation (beta range for two subscales=-.18 to .46)  (3) amount of clinical contact with older patients (beta=.19) | All groups:  (1) ethnicity  (2) future surgical residency preference  (3) exposure to geriatric health issues education | *Measurement:* Low reliability for measure. | More positive attitude scores were found in female respondents, and those reporting greater levels of intrinsic motivation as a reason for entering medicine. For 3^rd^ and 4^th^ years, positive attitudes were more likely in those reporting greater amounts of clinical contact with older patients. |
| De Biasio, Parkas & Soriano (2015) [15] | 404 medical students  (1MS-4MS), repeated cross section, United States  Response rate 38-83% | UCLA Geriatric Attitudes Scale [35] | (1) Year of med school (effect size n.r.)  (2) gender (effect size n.r.)  (3) age at start of med school (effect size n.r.) | (1) Race/ethnicity  (2) Undergraduate degree subject  (3) interest in social/  emotional or technical/  scientific topics | *Descriptives:* Reports of the individual participant year groups is unclear.  *Bias:* Vastly different response rates between groups; low response rate for some groups.  *Statistical:* No power estimation. | Females had more positive scores than males. Those over the age of 24 years at the start of med school had more positive scores than those who were younger |
| Edwards & Aldous (1996) [16] | 93 teaching faculty doctors, and 290 medical students (1MS, 3MS-5MS),  United Kingdom  Response rate 65-70% | Aging Semantic Differential [47] | (1) Knowledge score (effect size n.r.) | (1) Gender  (2) year of medical school  (3) attitude scores from students in same year group of a non-medicine-related course (English, and Computer science) | *Statistical:* Inappropriate statistical testing*;* no power estimation or justification of sample size; small sample. | Attitude scores were more positive in those with higher knowledge scores. |
| Fields, Jutagir, Adelman, Tideiksarr, & Olson (1992) [17] | 127 medical students  (4MS),  United States.  Response rate 100% | Aging Semantic Differential [47] | (1) Age (effect size n.r.) | (1) Gender  (2) knowledge scores  (3) specialty preference  (4) prior nursing home experience  (5) contact with elderly  (6) having previously completed gerontology coursework | *Statistical:* No power estimation or justification of sample size. | Younger medical students had more positive attitude scores. |
| Fitzgerald, Wray, Halter, Williams, & Supiano (2003) [18] | 171 medical students:  (1MS),  United States  Response rate 89% | UCLA Geriatric Attitudes Scale [35]  General attitudes category of Maxwell-Sullivan Attitude Survey [30] | (1) Gender (UCLA only) (effect size n.r.)  (2) interest in geriatric medicine career (beta=.28) | (1) Gender (MSAS only)  (2) Ethnicity  (3) patient age group preference  (4) prior care experience  (5) knowledge scores | *Measurement:* Low reliability for MSAS measure; no rationale for use of modified measure; validity of MSAS is unknown.  *Statistical:* No power estimation. | Attitude scores were more positive in females, and students reporting a greater interest in a geriatric medicine career. |
| Hellbusch, Corbin, Thorson, & Stacy (1995) [19] | 200 doctors, United States  Response rate 47% | Modified Kogan’s Attitudes Toward Old People Scale [48] | (1) Age (effect size n.r.)  (2) Number of years in practice (r=.19) | (1) Gender  (2) percent of patients over 65  (3) previous course on aging  (4) doctor specialty | *Statistical:* Inappropriate statistical testing.  *Descriptives:* Over 90% sample male without explanations provided.  *Instrumentation:* Failed to provide rationale for modifying measure. | Attitude scores were more positive in those with fewer years in practice. The oldest group of doctors had the most negative attitude scores. |
| Hogan, Chan, & Hansoti (2014) [20] | 173 doctors, United States  Response rate 60% | UCLA Geriatric Attitudes Scale [35] | n.r. | (1) Year of postgraduate training | *Bias:* Highly variable participation rates across sites.  *Measurement:* Low reliability for measure.  *Statistical:* No power estimation or justification of sample size. | Variables studied showed no relationship with attitude scores. |
| Hollar, Roberts, & Busby-Whitehead (2011) [21] | 116 medical students  (1MS), United States  Response rate 73% | UCLA Geriatric Attitudes Scale [35] | (1) Gender (effect size n.r.) | n.r. | *Statistical:* No power estimation. | Attitude scores were more positive in females. |
| Holtzman, Toewe II, & Beck (1979) [22] | 314 medical students  (1MS-4MS), United States  Response rate 48-98% | Aging Semantic Differential [47] | (1) Interest in primary care career (family, general internal and paediatrics) (effect size n.r.) | (1) Gender  (2) age  (3) year of medical school | *Descriptives:* Failed to provide sufficient information on sample demographics.  *Bias:* Vastly different response rates between groups.  *Statistical:* Inappropriate statistical testing no power estimation or justification of sample size. | Attitude scores were more positive in students expressing an interest in a primary care medicine career. |
| Holtzman, Beck, & Ettinger (1981) [23] | 118 medical students  (1MS & 3MS), United States  Response rate unclear | Aging Semantic Differential [47] | (1) Knowledge scores (for 3MS only). (r=.26) | (1) Year of medical school  (2) age | *Bias:* Did not address the group differences between 1MS and 3MS; did not provide response rates.  *Statistical:* No power estimation or justification of sample size. | Attitude scores were more positive in those with higher knowledge scores for the 3^rd^ year medical student group only. |
| Hughes et al. (2008) [24] | 163 medical students:  (1MS), United Kingdom  Response rate 96% | UCLA Geriatric Attitudes Scale [35] | (1) Willingness to consider geriatric medicine career (r=.39) | (1) Age  (2) gender  (3) ethnicity  (4) previous experience of caring for older people | *Measurement:* Low reliability for measure. | Attitude scores were more positive in those more willing to consider a career in geriatric medicine. |
| Kishimoto, Nagoshi, Williams, Masaki, & Blanchette (2005) [25] | 156 medical students (1MS-3MS) and 55 doctors, United States  Response rate 75-100% | UCLA Geriatric Attitudes Scale [35] | (1) Year in medical school or career (effect size n.r.) | n.r. | *Statistical:* Inappropriate statistical testing; description of variables tested unclear; no power estimation or justification of sample size. | Attitude scores were more positive in first year medical students and geriatrics fellows. |
| Lee, Reuben, & Farrell (2005) [26] | 177 doctors, United States  Response rate 84-97% | UCLA Geriatric Attitudes Scale [35] | (1) Personal experience with older people  (2) professional experience with older people  (3) interest in geriatric medicine career  (4) ethnicity  (5) Year of residency  (all effect sizes n.r.) | n.r. | *Descriptives:* Demographic information unclear.  *Statistical:* No power estimation or justification of sample size. | Attitude scores were more positive in those with greater personal and professional experience with older people, and those with a greater interest in geriatric medicine career. Asian-Americans had more negative attitude scores than Caucasians. Attitude scores were more positive in PGY2 than in PGY1 and PGY3. |
| Leung, LoGiudice, Schwarz, & Brand (2011) [27] | 122 doctors, Australia  Response rate 35-80% | Fraboni's Scale of Ageism [56] | (1) Age  (2) gender  (3) job seniority  (4) amount of social contact with older people  (5) interest in geriatric care  (all effect sizes n.r.) | (1) Country of birth  (2) languages spoken | *Participants:* Vastly difference response rates between groups; demographics of the groups unclear.  *Variables:* Inclusion of some variables was not justified. | Attitude scores were more positive in females, those over the age of 30 years, those with a more senior professional grade, and those with higher amounts of contact with healthy older people. |
| Linn & Zeppa (1988) [28] | 179 medical students (3MS), United States.  Response rate 98% | Kogan’s Attitudes Toward Old People Scale [48] | (1) Attitudes towards geriatric medicine career (r=.29)  (2) interest in family medicine career (r=36)  3) knowledge about aging (r=.38) | n.r. | *Measurement*: No justification given for modifying the attitude measure  *Statistical:* Unclear which variables were tested; no power estimation. |  |
| Lui & Wong (2009) [29] | 54 doctors, Singapore  Response rate 83% | Kogan’s Attitudes Toward Old People Scale [48] | (1) Seeing older patient care as ’unrewarding’ (effect size n.r.) | (1) Age  (2) marital status  (3) medical school attended  (4) nationality  (5) years in practice/seniority | *Measurement:* Did not justify inclusion of some of the variables; no justification of modifying the attitude measure.  *Statistical:* No power estimation or justification of sample size. | Attitude scores were more positive in doctors who reported older patient care as rewarding work. |
| Maxwell & Sullivan (1980) [30] | 150 doctors, United States  Response rate 38% | Maxwell Sullivan Attitudes Scale [30] | (1) Year in residency (effect size n.r.) | n.r. | *Participants*: Groups’ response rates unclear  *Bias:* Low response rate across sample.  *Measurement*: Did not perform factor analysis for factor validation; low reliability for measure.  *Statistical:* No power estimation or justification of sample size. | Attitude scores increased in positivity with each year of residency. |
| Menz, Stewart, & Oates (2003) [31] | 81 medical students (3MS & 4MS), Australia  Response rate unclear | Aging Semantic Differential [47]  Chumbler et al. attitude measure – Effectiveness subscale only [13] | (1) Knowledge scores (r=-29)  (2) gender (effect size n.r.) | (1) Intrinsic/  extrinsic motivational reasons for entering medicine  (2) age  (3) desire to work in private/public sector  (4) contact with grandparents | *Participants:* Response rate not reported; recruitment of sample unclear.  *Instrumentation:* Inappropriate scoring and analysis of the motivation measure.  *Bias:* Students were identifiable; sample had completed compulsory geriatrics course immediately prior to study.  *Statistical:* No power estimation or justification of sample size; small sample. | Attitude scores were more positive in females, and those with higher knowledge scores. |
| Muangpaisan, Intalapapron, & Assantachai (2008) [32] | 60 doctors and 146 medical students  (4MS), Thailand  Response rate 50-61% | UCLA Geriatric Attitudes Scale [35] | (1) Exposure to older people (r=.15) | (1) Gender  (2) age  (3) number of elderly people living at home | *Statistical:* Inappropriate statistical testing; no power estimation or justification of sample size; small sample (doctors)  *Participants:* Did not report how participants were recruited. | Attitude scores were more positive in those with greater exposure to older people. |
| Perrotta, Perkins, Schimpfhau-ser, & Calkins (1981) [33] | 127 medical students  (1MS), United States  Response rate 100% | Modified Kogan’s Attitudes Toward Old People Scale [48] | (1) Knowledge scores (r=-.43) | (1) Amount of contact with older people  (2) attitudes toward geriatric medicine | *Statistical:* Inappropriate statistical testing; no power estimation or justification of sample size. | Attitude scores were more positive in those with higher knowledge scores. |
| Reuben, Fullerton, Tschann, & Croughan-Minihane (1995) [34] | 554 medical students  (1MS), United States  Response rate 92% | Aging Semantic Differential [47]  Maxwell Sullivan Attitudes Scale [30] | (1) Gender  (2) ethnicity  (3) age  (4) knowledge scores  (all effect sizes n.r.) | (1) Marital status  (2) undergraduate degree  (3) age of oldest parent  (4) having taken a prior geriatrics courses  (5) prior work with older people | *No major issues identified* | Attitude scores were more positive in females, older respondents, and those with higher knowledge scores. Attitude scores were more negative in Asian-Americans. |
| Reuben et al. (1998) [35] | 142 doctors, United States  Response rate unclear | UCLA Geriatric Attitudes Scale [35] | (1) Year of residency  (2) job seniority  (3) interest in geriatric career  (all effect sizes n.r.) | (1) Specialty  (2) gender  (3) age  (4) ethnicity | *Participants*: Response rate data not provided.  *Statistical:* Statistical tests and analyses conducted not clearly reported; no power estimation or justification of sample size. | Attitude scores were more positive in PGY1-PGY3 doctors who reported an interest in pursuing geriatric medicine. Attitude scores were more positive in fellows and faculty members (teaching staff) than PGY doctors. Attitude scores were more negative in PGY1 and PGY2 doctors than other respondents. |
| Richter & Buck (1990) [36] | 85 doctors, United States  Response rate 69-86% | Maxwell-Sullivan Attitude Scale [30] | (1) Amount of geriatric-related didactic training and educational experience (in undergraduate school, medical school, and residency programme)  (2) faculty attitude scores.  (all effect sizes n.r.) | (1) Living parents older than 65  (2) contact with grandparents  (3) contact with elderly other than grandparents  (4) settings of educational experience with elderly  (5) year of residency | *Statistical:* No power estimation or justification of sample size; small sample.  *Descriptives:* Demographics information not fully reported. | PGY1-PGY attitude scores were more positive in those who had had a greater amount of didactic training in geriatrics. PGY1-PGY3 attitude scores showed a positive association with faculty attitude score. |
| Ruiz et al. (2015) [37] | 103 medical students  (1MS-4MS), United States  Response rate 14% | Fraboni's Scale of Ageism [56] | (1) Gender (Cohen’s d= .81)  (2) Internal motivation to respond without prejudice (Cohen’s d=1.41)  (3) Intention to work with older adults (Cohen’s d= .60) | (1) Year of med school  (2) Ethnicity | *Generalisability:* Very low response rate.  *Bias:* Questionnaire only offered in online format and may result in selection effects.  *Statistical:* No power estimation or justification of sample size. | Attitude scores were more positive in female medical students, those who intended to work with older adults and those who reported high internal high internal motivation to respond without prejudice. |
| Sainsbury, Wilkinson, & Smith (1994) [38] | 68 doctors, New Zealand  Response rate 62% | Aging Semantic Differential [47] | n.r. | (1) Year of residency  (2) having completed a health care of the elderly clinical attachment in 4th year | *Descriptives:* Demographic information not fully reported.  *Statistical:* No power estimation or justification of sample size; small sample. | Variables studied showed no relationship with attitude scores. |
| Shahidi & Devlen (1993) [39] | 84 medical students  (2MS), United Kingdom  Response rate unclear | Aging Semantic Differential [47] | (1) Knowledge scores (r=-.19) | (1) Gender  (2) age | *Participants:* Response rate data not reported; method of recruitment unclear.  *Statistical:* No power estimation or justification of sample size; small sample. | Attitude scores were more positive in those with higher knowledge scores. |
| Thorson & Powell (1991) [40] | 277 medical students  (1MS), United States  Response rate unclear | Kogan’s Attitudes Toward Old People Scale [48] | (1) Personality trait of dominance (r=-0.13) | (1) 14 of the 15 personality traits measured | *Participants:* Details of recruitment not reported; number in each cohort not reported; response rate not reported.  *Statistical:* No power estimation. | Attitude scores were more negative in respondents who had higher levels of the dominance personality trait. |
| Voogt, Mickus, Santiago, & Herman (2008) [41] | 231 medical students  (1MS), United States  Response rate 73-75% | UCLA Geriatrics Attitude Scale [35] | (1) Interest in geriatric medicine (beta=.33)  (2) amount of prior experiences caring for older adults (beta=.18)  (3) quality of relationships with older relatives (beta=.14) | (1) Age  (2) gender  (3) ethnicity  (4) frequency of interaction with older relatives | *Statistical:* power estimation and consideration of sample size unclear. | Attitude scores were more positive in those with a greater interest in pursuing a geriatric medicine career, those who had prior experiences of caring for older adults, and those who had a higher quality relationship with older relatives. |
| Wilderom et al. (1990) [42] | 663 medical students  (1MS) from six cohorts, United States  Response rate 82% | Kogan’s Attitudes Toward Old People Scale [48] | (1) Interest in geriatric medicine (r= .37)  (2) preference for treating elderly (r=.28)  (3) perception of elderly patients (r=.32)  (4) perceived physician attitude to elderly as patients (r=-.35)  (5) feeling of closeness with grandparents (r=.24)  (6) feeling of closeness with non-familial elderly (r=-.35)  (7) desired future community size (r=.18)  (8) voluntary work with elderly (r=-.25) | (1) Age  (2) gender  (3) aging-related undergraduate coursework  (4) perceived social skills  (5) desired residency choice | *Measurement:* Low reliability for measure reported  *Statistical:* No power estimation.  *Descriptives:* Sample characteristics unclear. | Attitude scores were more positive in those with a greater interest in pursuing a geriatric medicine career, those with greater feelings of closeness to grandparents and non-familial elderly, those who desired to work in a larger community in their future practice, those who had completed voluntary work with the elderly in the past, and those who reported a preference for treating older patients over younger patients. |
| Yang, Xiao, Ullah, & Deng (2013) [43] | 270 doctors, China  Response rate 67.5% | Chinese version of the Aging Semantic Differential [47]  Chinese version of Palmore’s Bias score FAQ1 [50] | (1) Gender (FAQ1 bias score only) (effect size n.r.)  (2) Knowledge scores (r=.16) | (1) Gender (ASD only)  (2) seniority  (3) age  (4) years of experience | *Bias:* Study may be affected by training/natural intervention.  *Generalisability:* Participant group derives from individuals who have self-selected to a national continuing medical education training course. | Male doctors showed higher negative bias scores than females using Palmore’s FAQ1, but no differences were found on the aging semantic differential. Lower knowledge scores was associated with lower attitude scores. |
| Zverev (2013) [44] | 154 Medical students (1MS-5MS) randomly selected from full class list, Malawi  Response rate 88% | Kogan’s Attitudes Toward Old People Scale [48] | n.r. | (1) Gender  (2) Year of study | *Sample:* Justification for sample size unclear. | Variables studied showed no relationship with attitude scores. |

†1MS-5MS denotes year in medical school from Year 1 to Year 5; n.r.‡ denotes that data was not reported

**Supplementary Appendix D:**

**Full reference list**

1. Oliver, D. and E. Burns, Geriatric medicine and geriatricians in the UK. How they relate to acute and general internal medicine and what the future might hold? Future Hosp J, 2016. 3(1): p. 49-54.

2. Oakley R., et al., Equipping tomorrow's doctors for the patients of today. Age Ageing, 2014. 43(4): p. 442-7.

3. Tullo, E.S., J. Spencer, and L. Allan, Systematic review: helping the young to understand the old. Teaching interventions in geriatrics to improve the knowledge, skills, and attitudes of undergraduate medical students. J Am Geriatr Soc, 2010. 58(10): p. 1987-1993.

4. Samra, R., et al., Changes in medical student and doctor attitudes toward older adults after an intervention: A systematic review. J Am Geriatr Soc, 2013. 61(7): p. 1188-1196.

5. Liu, Y., I.J. Norman, and A.E. While, Nurses' attitudes towards older people: A systematic review. Int J Nurs Stud, 2013. 50(9): p. 1271-1282.

6. Long, A., et al., Developing evidence based social care policy and practice. Part 3: Feasibility of undertaking systematic reviews in social care, Salford: University of Leeds. 2002.

7. von Elm, E., et al., The Strengthening the Reporting of Observational Studies in Epidemiology (STROBE) statement: guidelines for reporting observational studies. Lancet, 2007. 370(9596): p. 1453-1457.

8. Beall, C., et al., Teaching geriatrics medicine: residents’ perceptions of barriers and stereotypes. Gerontol Geriatr Educ 1991; 85-96

9. Belgrave, L.L., et al., Stereotyping of the aged by medical students. Gerontol Geriatr Educ, 1982. 3(1): p. 37-44.

10. Cammer Paris, B.E., et al., First year medical student attitudes toward the elderly: A comparison of years 1986, 1991 and 1994. Gerontol Geriatr Educ, 1997. 18(1): p. 13-22.

11. Cheong, S.K., T.Y. Wong, and G.C.H. Koh, Attitudes towards the elderly among Singapore medical students. Ann Acad Med Singapore, 2009. 38(10): p. 857-861.

12. Chua, M.P.W., et al., Attitudes of First-year Medical Students in Singapore Towards Older People and Willingness to Consider a Career in Geriatric Medicine. Ann Acad Med Singapore, 2008. 37(11): p. 947-951.

13. Chumbler, N.R., J.M. Robbins, and M.E. Poplawski, Rewards of entering pediatric medicine and attitudes toward older adults. J Am Podiatr Med Assoc, 1996. 86(6): p. 288-294.

14. Chumbler, N.R. and T.E. Ford, The orientation of health professional students towards the care of older adults: The case of podiatry. Health, 1998. 2(3): p. 259-281.

15. De Biasio, J.C., V. Parkas, and R.P. Soriano, Longitudinal assessment of medical student attitudes toward older people. Med Teach, 2016. 38(8): p. 823-828.

16. Edwards, M.J.J. and I.R. Aldous, Attitudes to and knowledge about elderly people: A comparative analysis of students of medicine, English and computer science and their teachers. Med Educ, 1996. 30(3): p. 221-225.

17. Fields, S.D., et al., Geriatric education 1: Efficacy of a mandatory clinical rotation for 4th year medical students. J Am Geriatr Soc, 1992. 40(9): p. 964-969.

18. Fitzgerald, J.T., et al., Relating Medical Students' Knowledge, Attitudes, and Experience to an Interest in Geriatric Medicine. Gerontologist, 2003. 43(6): p. 849-855.

19. Hellbusch, J.S., et al., Physicians' attitudes towards aging. Gerontol Geriatr Educ, 1995. 15(2): p. 55-65.

20. Hogan TM, Chan SB, and Hansoti B, Multidimensional attitudes of emergency medicine residents toward older adults. West J Emerg Med, 2014. 15(4): p. 511-517.

21. Hollar, D., E. Roberts, and J. Busby-Whitehead, COCOA: A new validated instrument to assess medical students' attitudes towards older adults. Educ Gerontol, 2011. 37(3): p. 193-209.

22. Holtzman, J.M., C.H. Toewe II, and J.D. Beck, Specialty preference and attitudes toward the aged. J Fam Pract, 1979. 9(4): p. 667-672.

23. Holtzman, J.M., J.D. Beck, and R.L. Ettinger, Cognitive knowledge and attitudes toward the aged of dental and medical students. Educ Gerontol, 1981. 6(2-3): p. 195-207.

24. Hughes, N.J., et al., Medical student attitudes toward older people and willingness to consider a career in geriatric medicine. J Am Geriatr Soc, 2008. 56(2): p. 334-338.

25. Kishimoto, M., et al., Knowledge and Attitudes About Geriatrics of Medical Students, Internal Medicine Residents, and Geriatric Medicine Fellows. J Am Geriatr Soc, 2005. 53(1): p. 99-102.

26. Lee, M., D.B. Reuben, and B.A. Ferrell, Multidimensional attitudes of medical residents and geriatrics fellows toward older people. J Am Geriatr Soc, 2005. 53(3): p. 489-494.

27. Leung, S., et al., Hospital doctors' attitudes towards older people. Internal Med J, 2011. 41(4): p. 308-314.

28. Linn, B.S. and R. Zeppa, Predicting third year medical students' attitudes toward the elderly and treating the old. Gerontol Geriatr Educ, 1988. 7(3-4): p. 167-175.

29. Lui, N.L. and C.H. Wong, Junior doctors' attitudes towards older adults and its correlates in a tertiary-care public hospital. Ann Acad Med Singapore, 2009. 38(2): p. 125-129.

30. Maxwell, A.J. and N. Sullivan, Attitudes toward the geriatric patient among family practice residents. J Am Geriatr Soc, 1980. 28(8): p. 341-345.

31. Menz, H.B., F.A. Stewart, and M.J. Oates, Knowledge of aging and attitudes toward older people - A survey of Australian podiatric medical students. J Am Podiatr Med Assoc, 2003. 93(1): p. 11-17.

32. Muangpaisan, W., S. Intalapapron, and P. Assantachai, Attitudes of medical students and residents toward care of the elderly. Educ Gerontol, 2008. 34(5): p. 400-406.

33. Perrotta, P., et al., Medical student attitudes toward geriatric medicine and patients. J Med Educ, 1981. 56(6): p. 478-483.

34. Reuben, D.B., et al., Attitudes of beginning medical students toward older persons: a five-campus study. The University of California Academic Geriatric Resource Program Student Survey Research Group. J Am Geriatr Soc, 1995. 43(12): p. 1430-1436.

35. Reuben, D.B., et al., Development and validation of a geriatrics attitudes scale for primary care residents. J Am Geriatr Soc, 1998. 46(11): p. 1425-1430.

36. Richter, R.C. and E.L. Buck, Family practice residents and the elderly: fostering positive attitudes. Fam Med, 1990. 22(5): p. 388-391.

37. Ruiz, J.G., et al., Group-based differences in anti-aging bias among medical students. Gerontol Geriatr Educ, 2015. 36(1): p. 58-78.

38. Sainsbury, R., T.J. Wilkinson, and C.W. Smith, Do the clinical years change medical students' attitudes to old people? Med Educ, 1994. 28(4): p. 307-311.

39. Shahidi, S. and J. Devlen, Medical students' attitudes to and knowledge of the aged. Med Educ, 1993. 27(3): p. 286-288.

40. Thorson, J.A. and F.C. Powell, Medical students' attitudes towards ageing and death: A cross-sequential study. Med Educ, 1991. 25(1): p. 32-37.

41. Voogt, S., et al., Attitudes, Experiences, and Interest in Geriatrics of First-Year Allopathic and Osteopathic Medical Students. J Am Geriatr Soc, 2008. 56(2): p. 339-344.

42. Wilderom, C.P.M., et al., Correlates of entering medical students' attitudes toward geriatrics. Educ Gerontol, 1990. 16(5): p. 429-446.

43. Yang, Y.N., et al., General practitioners' knowledge of ageing and attitudes towards older people in China. Australasian Journal on Ageing, 2015. 34(2): p. 82-87.

44. Zverev, Y., Attitude towards older people among Malawian medical and nursing students. Educ Gerontol, 2013. 39(1): p. 57-66.

45. Murphy, K.R., B. Myors, and A.H. Wolach, Statistical Power Analysis: A Simple and General Model for Traditional and Modern Hypothesis Tests, 2009, Oxon, UK: Routledge.

46. Schönbrodt, F. and M. Perugini, At what sample size do correlations stabilize? Journal of Research in Personality, 2013. 47(5): p. 609-12.

47. Rosencranz, H.A. and T.E. McNevin, A factor analysis of attitudes toward the aged. Gerontologist, 1969. 9(1): p. 55-59.

48. Kogan, N., Attitudes toward old people: The development of a scale and an examination of correlates. J Abnorm Soc Psychol, 1961. 62(1): p. 44-54.

49. Hilt, M.L., The Kogan Attitudes Toward Old People scale: Is it time for a revision? Psychol Rep, 1997. 80(3): p. 1372-1374.

50. Palmore, E., Facts on aging: A short quiz. Gerontologist, 1977. 17(4): p. 315-320.

51. Palmore, E., The facts on aging quiz: Part two. Gerontologist, 1981. 21(4): p. 431-437

52. Lee M, et al., Development and validation of a geriatric knowledge test for medical students. Journal of the American Geriatrics Society, 2004. 52(6): p. 983-8.

53. Merrill, J.M., et al., Measuring social desirability among senior medical students. Psychol Rep, 1995. 77(3 Pt 1): p. 859-864.

54. Crandall SJ, Volk RJ, and V. Loemker, Medical students' attitudes toward providing care for the underserved: are we training socially responsible physicians? JAMA, 1993. 269(19): p. 2519-23.

55. Merrill J, et al., Uncertainties and ambiguities: measuring how medical students cope. Med Educ., 1994. 28(4): p. 316-322.

56. Ghosh AK, On the challenges of using evidence-based information: the role of clinical uncertainty. Journal of Laboratory and Clinical Medicine, 2004. 144(2): p. 60-64.

57. Simpkin AL and Schwartzstein RM, Tolerating Uncertainty—The Next Medical Revolution? New England Journal of Medicine, 2016. 375(18): p. 1713-1715.

58. Tsugawa Y, et al., Comparison of Hospital Mortality and Readmission Rates for Medicare Patients Treated by Male vs Female Physicians. JAMA Intern Med, 2016. Published online December 19, 2016. doi:10.1001/jamainternmed.2016.7875.

59. Fraboni, M., R. Saltstone, and S. Hughes, The Fraboni scale of ageism: An attempt at a more precise measure of ageism. Can J Aging, 1990. 9(1): p. 56-66.
